# Global collaborative networks on meta-analyses of randomized trials published in high impact factor medical journals: a social network analysis

**DOI:** 10.1186/1741-7015-12-15

**Published:** 2014-01-29

**Authors:** Ferrán Catalá-López, Adolfo Alonso-Arroyo, Brian Hutton, Rafael Aleixandre-Benavent, David Moher

**Affiliations:** 1 Division of Pharmacoepidemiology and Pharmacovigilance, Spanish Medicines and Healthcare Products Agency (AEMPS), Madrid, Spain; 2 Fundación Instituto de Investigación en Servicios de Salud, Valencia, Spain; 3 López Piñero Institute for the History of Medicine and Science (IHMC), Spanish National Research Council (CSIC) and University of Valencia, Valencia, Spain; 4 Department of History of Science and Documentation, University of Valencia, Valencia, Spain; 5 Clinical Epidemiology Program, Ottawa Hospital Research Institute (OHRI), Ottawa, ON, Canada

**Keywords:** Authorship, Evidence-based medicine, Meta-analysis, Randomized controlled trial, Scientific collaboration, Social network analysis

## Abstract

**Background:**

Research collaboration contributes to the advancement of knowledge by exploiting the results of scientific efforts more efficiently, but the global patterns of collaboration on meta-analysis are unknown. The purpose of this research was to describe and characterize the global collaborative patterns in meta-analyses of randomized trials published in high impact factor medical journals over the past three decades.

**Methods:**

This was a cross-sectional, social network analysis. We searched PubMed for relevant meta-analyses of randomized trials published up to December 2012. We selected meta-analyses (including at least randomized trials as primary evidence source) published in the top seven high impact factor general medical journals (according to Journal Citation Reports 2011): *The New England Journal of Medicine*, *The Lancet*, the *BMJ*, *JAMA*, *Annals of Internal Medicine*, *Archives of Internal Medicine* (now renamed *JAMA Internal Medicine*), and *PLoS Medicine*. Opinion articles, conceptual papers, narrative reviews, reviews without meta-analysis, reviews of reviews, and other study designs were excluded.

**Results:**

Overall, we included 736 meta-analyses, in which 3,178 authors, 891 institutions, and 51 countries participated. The *BMJ* was the journal that published the greatest number of articles (39%), followed by *The Lancet* (18%), *JAMA* (15%) and the *Archives of Internal Medicine* (15%). The USA, the UK, and Canada headed the absolute global productivity ranking in number of papers. The 64 authors and the 39 institutions with the highest publication rates were identified. We also found 82 clusters of authors (one group with 55 members and one group with 54 members) and 19 clusters of institutions (one major group with 76 members). The most prolific authors were mainly affiliated with the University of Oxford (UK), McMaster University (Canada), and the University of Bern (Switzerland).

**Conclusions:**

Our analysis identified networks of authors, institutions and countries publishing meta-analyses of randomized trials in high impact medical journals. This valuable information may be used to strengthen scientific capacity for collaboration and to help to promote a global agenda for future research of excellence.

## Background

The past decades have seen the establishment of evidence synthesis, particularly systematic reviews and meta-analyses, as a key component of evidence based medicine (EBM) [1,2]. Meta-analyses of randomized trials have become more widely accepted by clinicians, researchers and policy makers as a useful tool to critically assess the totality of evidence in a research question. When performed well and reported completely, incorporating explicit and detailed methods and results, such studies produce information that can have undoubtedly major, immediate effects on medical practice, research agendas and the establishment of healthcare policies.

Important milestones that may have encouraged research in this field, from the point of view of scientific publications and institutional development of EBM [1-4], include the creation of international research groups, centers, and consortia (such as the Centre for Evidence Based Medicine and The Cochrane Collaboration in the 1990s) in addition to groups developing reporting guidelines to ensure articles contain all essential information, such as QUOROM (Quality of Reporting of Meta-analyses) [5] and, more recently, PRISMA (Preferred Reporting Items for Systematic Reviews and Meta-Analyses) [6].

Global health challenges require research collaboration and multi-lateral programs on a global scale, owing to the nature and magnitude of the public health problems. Noteworthy examples include major environmental, political, and social determinants of health, as well as complex and changing clinical issues related to the conditions and risk factors that cause the highest burden of disease around the world [7-9]. Despite continuous efforts of individual scientists and institutions to remedy deficiencies in healthcare effectiveness and safety, multiple gaps and disparities remain. Research collaboration contributes to the advancement of knowledge by exploiting the results of scientific efforts more efficiently, but the global patterns of collaboration on meta-analysis are unknown. Given that meta-analyses can provide high-quality clinical evidence regarding the robustness of the effects of healthcare interventions to inform medical practice, there is an urgent need to evaluate and promote scientific activity and growth in the field of EBM [4,10-12].

Social network analysis [13], the study of structure derived from the regularities in the patterning of relationships between social entities (which might be people or organizations), is grounded in the assessment of empirical data, and can provide an appropriate approach to identify top scientists and researchers, groups of excellence, and leading institutions. It also offers information to assess the citation patterns among papers within a specialty [14], to identify gaps in the evidence from scientific research [15], and to understand the structure and nature of relationships and interactions within a scientific community that collaborate to better achieve common or compatible goals [13,16].

This study aimed to describe and characterize global collaborative patterns with regard to the conduct of meta-analyses of randomized trials published over the past three decades in high impact factor medical journals, by applying techniques from social network analysis.

## Methods

### Design and sample

In December 2012, we searched for reports of meta-analyses of randomized trials that were indexed in PubMed and published in one of the top seven high impact general medical journals, as identified in 2011, based on an impact factor of at least 10 (subject categories ‘Medicine, General & Internal’ of Journal Citation Reports, Thomson Reuters): *The New England Journal of Medicine* (*NEJM*), *The Lancet*, the *British Medical Journal* (*BMJ*), the *Journal of the American Medical Association* (*JAMA*), *Annals of Internal Medicine*, *Archives of Internal Medicine* (now renamed *JAMA Internal Medicine*) and *PLoS Medicine*. Specifically, the following terms were used for PubMed: (‘meta-analysis’[Publication Type] OR ‘meta-analysis as topic’[MeSH Terms] OR ‘meta-analysis’[All Fields]) AND (‘randomized controlled trial’[Publication Type] OR ‘randomized controlled trials as topic’[MeSH Terms] OR ‘randomized controlled trial’[All Fields]) AND (‘Lancet’[Journal] OR ‘N Engl J Med’[Journal] OR ‘JAMA’[Journal] OR ‘Br Med J’[Journal] OR ‘Br Med J (Clin Res Ed)’[Journal] OR ‘BMJ’[Journal] OR ‘PLoS Med’[Journal] OR ‘Ann Intern Med’[Journal] OR ‘Arch Intern Med’[Journal]) AND (hasabstract[text] AND ‘humans’[MeSH Terms]). We also performed complementary hand-searches and reviewed references of identified eligible reports to identify additional meta-analyses.

We included two types of articles from the eligible journals: original research reports and reviews (both incorporating meta-analyses of randomized trials). Editorials, commentaries, and other opinion articles were excluded. We also excluded conceptual papers, literature (narrative) reviews, reviews of reviews, meta-analysis of observational studies not considering randomized trials, single randomized trials, and other study designs (such as cost-effectiveness analyses and epidemiological studies).

For the purposes of this study, we selected all articles published in English and indexed in PubMed between January 1985 and December 2012. One researcher with expertise in evidence synthesis (FC-L) screened the titles and abstracts, and identified all potentially eligible articles. The same researcher excluded the articles not meeting the pre-specified criteria.

### Data extraction

For each included paper, we extracted information on the year of publication, the journal title, and the authors’ names, institutional affiliation(s), and country of origin. This information was downloaded online through the Science Citation Index-Expanded (SCI-E) Web of Knowledge platform version 5, in April 2013. The Web of Knowledge platform is a database that contains all the above information, including the full addresses of all authors of every paper. We also used the SCI-E to determine the extent to which each study had been cited in the scientific peer-review literature using the ‘times cited’ number (that is, the number of times a publication has been cited by other publications). A process of standardization was conducted to bring together the different names of a particular author or institution. Specifically, one researcher (AA-A) checked the names by which an individual author appeared in two or more different forms (for example, ‘Gordon Guyatt’ or ‘Gordon H Guyatt’), using coincidence in that author’s place(s) of work as the basic criterion for normalization (for example, McMaster University, Canada). In the case of institutions, we unified the different variants to match the name recorded in public directories of institutions. Similarly, given that institutional names in many records included two or more institutions (for example, university hospitals, research centers and academic institutions), we proceeded to distinguish between these names by recording all variations of any individual macroinstitution as could be identified for each bibliographic record (for example, for the institutional address ‘Reproductive Medicine Unit, Department of Obstetrics & Gynaecology, University of Adelaide, Queen Elizabeth Hospital, Australia’, the standardization approach was to present ‘University of Adelaide, Australia’ separately from ‘Queen Elizabeth Hospital, Australia’). With all this information, we constructed a Microsoft Access database.

### Data analysis

In this paper, we use the term ‘co-authorship’ to refer to joint authorship of a scientific paper by at least two individuals, and the term ‘institutional collaboration’ to refer to joint authorship by different institutions. ‘Intensity of collaboration or threshold’ refers to the number used to form clusters of authors and institutions (that is, the frequency of co-authorship between pairs of authors or of collaboration between institutions), and reflects a criterion to label identifiable clusters as research groups. Collaboration between authors (or institutions) was portrayed by calculating the number of papers, names, signatures and collaborations, the index of signatures per paper or collaboration index (which is the mean number of signatures per paper), and the index of authors per paper (mean number of authors per paper, considering only the different authors). A summary box with definitions of each of the measurements of collaboration is provided in the supplementary material (see Additional file 1: ‘Definitions of collaborative measurements’).

To construct co-authorship networks, we identified all combinations of pairs of authors for each paper. The number of co-authorships for each paper is related to the number of authors as is equal to

$$\frac{m!}{\left(m-n\right)!n!},$$

where *m* is the number of individual authors and *n* the number of elements in the groups constructed. Once co-authorship was quantified, we further established an *a posteriori* threshold of two or more collaborations between pairs of authors, in order to reduce the number of nodes and links that would prevent a clear view of the network, and thus center the analysis on the more intense co-authorship relationships. The same approach was applied to institutional and country authorship to construct the network of collaborations, although in this case, we applied an *a posteriori* threshold of three or more papers signed in co-authorship. The productivity and patterns of collaboration by author, institution and country were analyzed.

We used PAJEK [17], a software package for large network analysis that is free for non-commercial use, to analyze indicators and construct social networks.

## Results

### Number of meta-analyses

**Figure 1:**
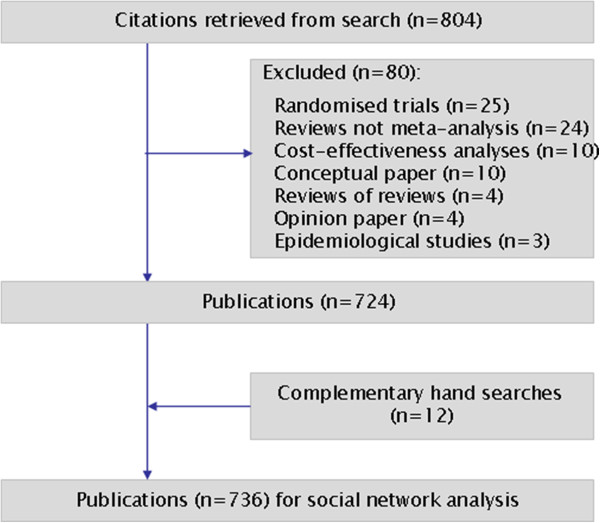
**Selection of publications.** Flow chart.

The PubMed search generated 804 records. Following screening of abstracts and full text articles, 724 publications were retained, and 12 additional publications were added from complementary searches of reference lists, thereby yielding a final sample of 736 included meta-analyses. The process of study selection is presented in Figure 1.

### General characteristics of the sample

The *BMJ* published the largest number of articles (n = 289; 39%), followed by *The Lancet* (n = 132; 18%), *JAMA* (n = 113; 15%) and the *Archives of Internal Medicine* (n = 112; 15%). Overall, 736 meta-analyses received 130,644 citations, of which 37,930 citations (29%) corresponded to *BMJ*, 34 911(27%) to *The Lancet* and 25,273 (19%) to *JAMA*. The number of publications increased exponentially over the study period (Table 1). Approximately three-quarters of the meta-analyses were reported during the most recent decade.

**Table 1:** General characteristics of the sample of study

**Characteristic**	**Number (%)**
Total number of articles	736 (100)
Articles per journal^a^	
*BMJ*	289 (39.3)
*The Lancet*	132 (17.9)
*JAMA*	113 (15.3)
*Arch Intern Med*	112 (15.2)
*Ann Intern Med*	70 (9.5)
*New Engl J Med*	13 (1.8)
*PLoS Med*	7 (0.9)
Citations per journal^b^	
*BMJ*	37,930 (29.0)
*The Lancet*	34,911 (26.7)
*JAMA*	25,273 (19.3)
*Ann Intern Med*	13,381 (10.2)
*Arch Intern Med*	11,945 (9.1)
*New Engl J Med*	6,992 (5.3)
*PLoS Med*	212 (0.1)
Articles per period of publication	
1985 to 1989	3 (0.4)
1990 to 1994	53 (7.2)
1995 to 1999	140 (19.0)
2000 to 2004	166 (22.6)
2005 to 2009	231 (31.4)
2010 to 2012	143 (19.4)
Country of publication (first author)^c^	
USA	214 (29.1)
UK	207 (28.1)
Canada	94 (12.8)
Australia	36 (4.9)
Switzerland	26 (3.5)
France	26 (3.5)
Denmark	20 (2.7)
Italy	20 (2.7)
Number of authors per paper	
1	41 (5.6)
2 to 3	166 (22.5)
4 to 6	297 (40.3)
> 7	232 (31.5)

^a^PLOS Medicine has been published weekly online since 19 October 2004.

^b^Information refers to the total citations received by all the papers published in a given journal (for example, 289 papers published in BMJ received a total of 37,930 citations).

^c^Information was incomplete for some records. We used country data of the first author in 704 out of 736 papers, while for 32 papers this information was retrieved from the correspondence address.

The included meta-analyses had a median of 5 authors, although 41 (6%) were single authored (Table 1). More than a quarter (214 [29%]) of the first authors were from the USA, with three countries (the USA, the UK and Canada) accounting for more than two-thirds of the meta-analyses published during the period of analysis (Table 1).

### Production and collaboration patterns

**Figure 2:**
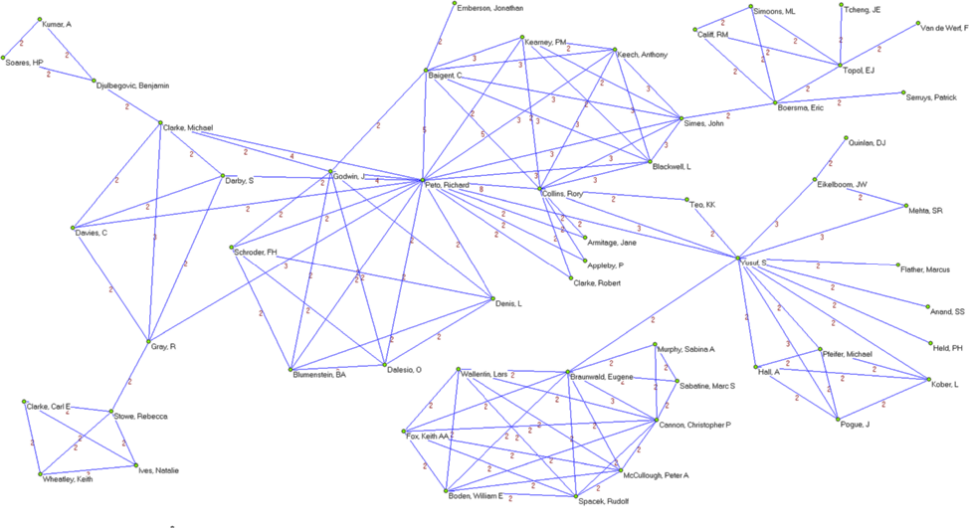
**Co-authorship networks.** Most productive cluster of authors, applying a threshold of two or more papers signed in co-authorship.

**Figure 3:**
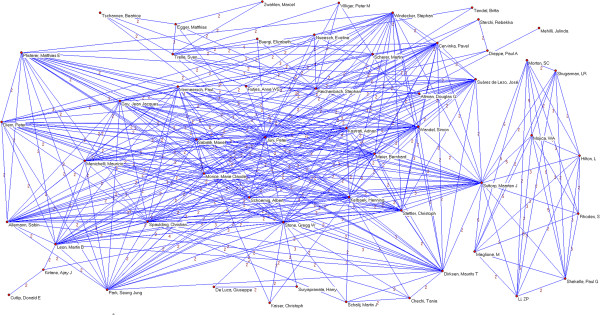
**Co-authorship networks.** Second most productive cluster of authors, applying a threshold of two or more papers signed in co-authorship.

**Figure 4:**
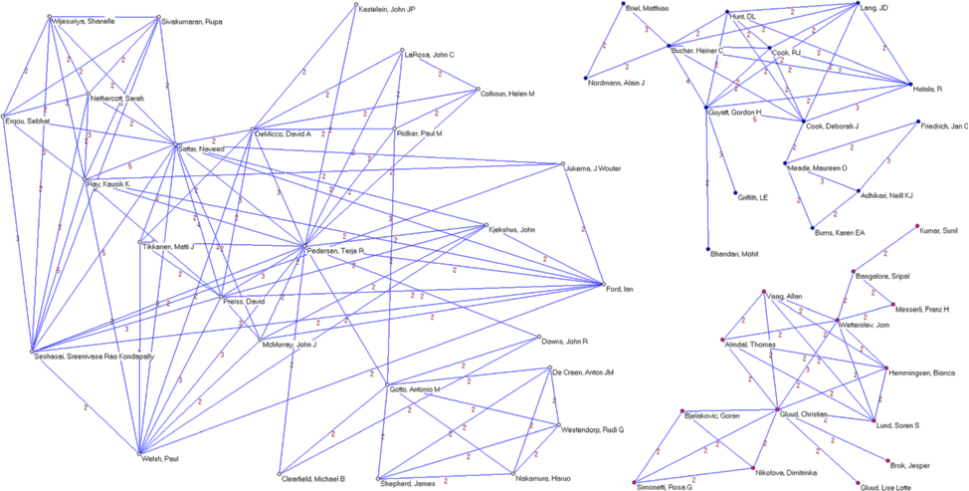
**Co-authorship networks.** Main clusters of authors (≥ 15 members), applying a threshold of two or more papers signed in co-authorship.

**Figure 5:**
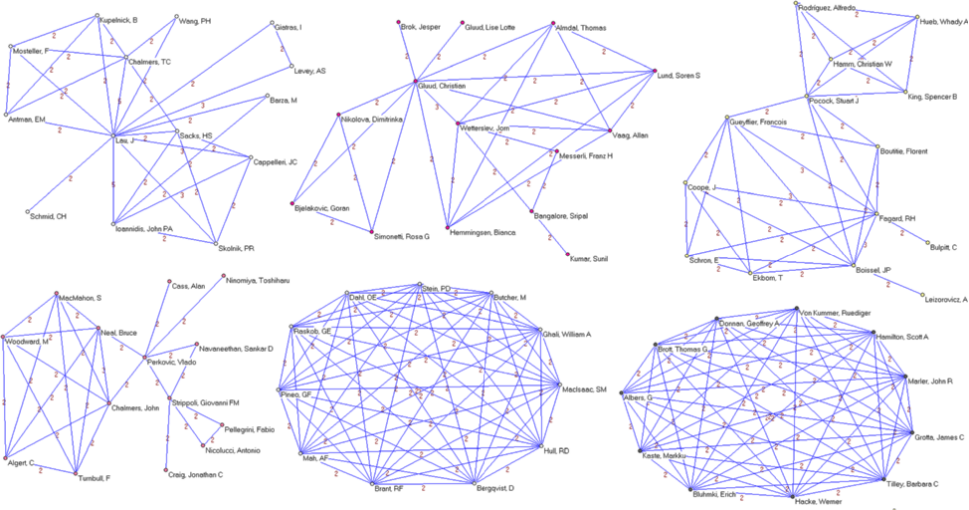
**Co-authorship networks.** Main clusters of authors (≤ 14 members), applying a threshold of two or more papers signed in co-authorship.

**Figure 6:**
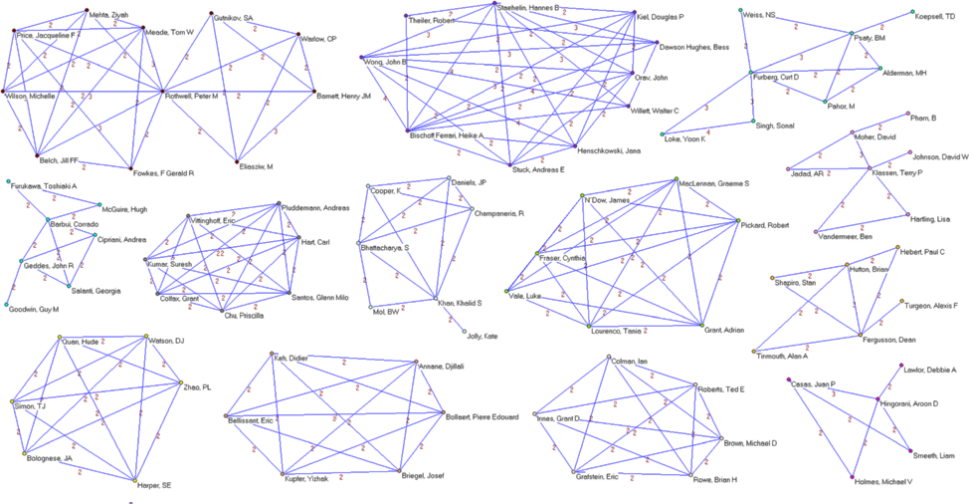
**Co-authorship networks.** Main clusters of authors (≤ 11 members), applying a threshold of two or more papers signed in co-authorship.

**Figure 7:**
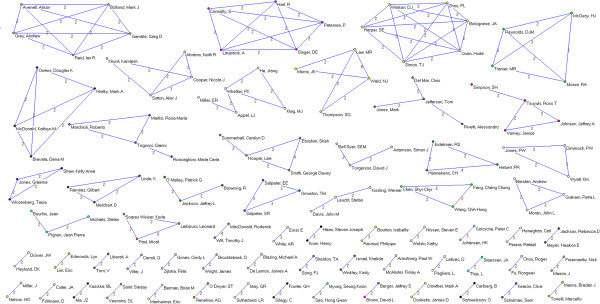
**Co-authorship networks.** Main clusters of authors (≤ 6 members), applying a threshold of two or more papers signed in co-authorship.

Overall, 3,178 authors, 891 institutions and 51 countries worldwide were involved in the sample of articles. We identified 64 authors who published 5 or more papers (Table 2). The most prolific authors were Lau (15 papers), Guyatt (14), Peto (13), Yusuf (12), Cook (11) and Jüni (11). Many of the most prolific authors are affiliated with only a few academic institutions and/or medical centers; six are affiliated with the University of Oxford (Peto, Collins, Clarke, Baigent, Gray and Rothwell), five are affiliated with McMaster University (Guyatt, Yusuf, Cook, Douketis, and Eikelboom) and five are affiliated with the University of Bern and/or the Inselspital – Bern University Hospital (Jüni, Trelle, Egger, Reichenbach and Nüesch). Applying a threshold of 2 or more papers published as co-authors (Figures 2, 3, 4, 5, 6, 7), we identified 82 clusters of authors. Of these, 12 were identified as major co-authorship groups (1 with 55 members, 1 with 54 members, 1 with 27 members, 1 with 15, 4 with 14 members, 3 with 11 members, 1 with 10 members, 1 with 8 members, 5 with 7 members, 5 with 6 members and 3 with 5 members).

**Table 2:** Ranking of most prolific authors (five or more papers) and their collaborative patterns

**Author**	**Primary affiliation at the time of publication**	**Year of first eligible paper**	**Total papers, n**	**Signatures, n**	**Collaborations, n**	**Main collaborators (number of papers)**
Lau, Joseph	Brown University, USA^a^	1992	15	90	54	Chalmers TC and Ioannidis JPA (5)
Guyatt, Gordon H	McMaster University, Canada	1996	14	104	76	Cook DJ (5)
Peto, Richard	University of Oxford, UK	1991	13	124	75	Collins R (8)
Yusuf, Salim	McMaster University, Canada	1989	12	83	54	Collins R, Eikelboom JW, Mehta SR and Pogue J (3)
Cook, Deborah J	McMaster University, Canada	1995	11	71	49	Guyatt GH (5)
Jüni, Peter	University of Bern, Switzerland	1996	11	124	59	Reichenbach S (8)
Collins, Rory	University of Oxford, UK	1991	9	78	45	Peto R (8)
Trelle, Sven	University of Bern, Switzerland	2007	9	103	67	Jüni P and Reichenbach S (7)
Bucher, Heiner C	University Hospital Basel, Switzerland	1996	8	60	41	Guyatt GH (4)
Egger, Mathias	University of Bern, Switzerland	2001	8	65	49	Jüni P, Reichenbach S and Trelle S (3)
Gluud, Christian	Copenhagen University Hospital, Denmark	2001	8	40	21	Wetterslev J (3)
Gøtzsche, Peter C	The Nordic Cochrane Centre, Denmark	1995	8	27	17	Johansen HK (3)
Jackson, Jeffrey L	Zablocki VA Medical Center, USA	1997	8	33	23	Browning R and O’Malley Patrick G (2)
Law, Malcolm R	Queen Mary University of London, UK	1991	8	24	7	Wald NJ (8)
Reichenbach, Stephan	University of Bern, Switzerland	2004	8	81	44	Jüni P (8)
Sutton, Alex J	University of Leicester, UK	2003	8	40	28	Cooper NJ (3)
Suttorp, Maarten J	St Antonius Hospital, the Netherlands	2003	8	120	61	Maglione M, Mojica WA, Morton SC and Shekelle PG (5)
Wald, Nicholas J	Queen Mary University of London, UK	1991	8	24	7	Law MR (8)
Clarke, Michael	University of Oxford, UK	1994	7	83	67	Peto R (4)
Furberg, Curt D	Wake Forest University School of Medicine, USA	1989	7	36	20	Loke YK, Psaty BM and Singh S (3)
Baigent, Colin	University of Oxford, UK	1996	6	50	26	Collins R and Peto R (5)
Boersma, Eric	Erasmus Medical Center, the Netherlands	2001	6	74	63	Califf RM, Serruys P, Simes J, Simoons ML and Topol EJ (2)
Boissel, Jean-Pierre	Université Claude Bernard Lyon 1, France	1992	6	42	26	Fagard RH and Gueyffier F (3)
Chalmers, Thomas C	Tufts Medical Center, Tufts University, USA	1990	6	31	16	Lau J (5)
Douketis, James D	McMaster University, Canada	2000	6	32	25	Crowther MA (2)
Ebrahim, Shah	London School of Hygiene and Tropical Medicine, UK	1997	6	70	60	Smith GD (4)
Ioannidis, John PA	Stanford University, USA	1995	6	32	17	Lau J (5)
Khan, Khalid S	Queen Mary University of London, UK	1996	6	39	27	Bhattacharya S, Champaneria R, Cooper K, Jolly K and Middleton LJ (2)
Klassen, Terry P	University of Manitoba, Canada	1996	6	48	36	Moher D (3)
Loke, Yoon K	University of East Anglia, UK	2000	6	18	7	Singh S (4)
McAlister, Finlay A	University of Alberta, Canada	2001	6	30	23	Armstrong PW (2)
Moher, David	Ottawa Hospital Research Institute, Canada	1996	6	52	42	Klassen TP (3)
Roberts, Ian	London School of Hygiene and Tropical Medicine, UK	1996	6	18	12	All co-authorship (1)
Sattar, Naveed	University of Glasgow, UK	2009	6	91	58	Ray KK and Seshasai SRK (5)
Shekelle, Paul G	Rand Corporation, USA	2003	6	57	29	Maglione M, Mojica WA, Morton SC and Suttorp MJ (5)
Stone, Gregg W	Columbia University Medical Center, USA	2005	6	120	77	Leon MB (4)
Tognoni, Gianni	Consorzio Mario Negri Sud, Italy	1993	6	78	69	Marchioli R, Marfisi RM and Roncaglioni MC (2)
Topol, Eric J	Scripps Translational Science Institute, USA	1991	6	44	33	Boersma E, Califf RM, Simoons ML, Tcheng JE and Van de Werf F (2)
Bischoff Ferrari, Heike A	University Hospital Zurich, Switzerland	2004	5	48	26	Dawson Hughes B, Staehelin HB, Willett WC and Wong JB (4)
Briel, Matthias	University Hospital Basel, Switzerland	2006	5	51	43	Bucher HC (3)
Eikelboom, John W	McMaster University, Canada	2000	5	19	10	Yusuf S (3)
Ernst, Edzard	University of Exeter, UK	1995	5	18	11	White AR (3)
Fagard, Robert H	University of Leuven, Belgium	1997	5	51	36	Boissel JP and Gueyffier F (3)
Fergusson, Dean	Ottawa Hospital Research Institute, Canada	1998	5	33	22	Hutton B (3)
Glasziou, Paul	Bond University, Australia	1993	5	26	21	All co-authorship (1)
Godwin, Jon	Glasgow Caledonian University, UK	1995	5	52	38	Peto R (4)
Gray, Richard	University of Oxford, UK	2001	5	48	36	Clarke M and Peto R (3)
Hennekens, Charles H	Florida Atlantic University, USA	1995	5	28	19	Hebert, PR (4)
Leibovici, Leonard	Rabin Medical Center, Israel	2003	5	22	12	Paul M (5)
Maglione, Margaret	Rand Corporation, USA	2003	5	49	22	Mojica WA, Morton SC, Shekelle PG and Suttorp MJ (5)
Mojica, Walter A	Rand Corporation, USA	2003	5	49	22	Maglione M, Morton SC, Shekelle PG, and Suttorp MJ (5)
Morton, Sally C	University of Pittsburgh, USA	2003	5	49	22	Maglione M, Mojica WA, Shekelle PG and Suttorp MJ (5)
Nüesch, Eveline	University of Bern, Switzerland	2009	5	35	16	Jüni P (5)
Paul, Mical	Rabin Medical Center, Beilinson Hospital, Israel	2003	5	22	12	Leibovici L (5)
Pignon, Jean-Pierre	Institut Gustave-Roussy, France	1999	5	53	46	Bourhis J and Michiels S (2)
Pocock, Stuart J	London School of Hygiene and Tropical Medicine, UK	1995	5	67	54	Boissel JP, Boutitie F, Fagard RH, Gueyffier F, Hamm CW, Hueb WA, King SB and Rodríguez A (2)
Ray, Kausik K	St George’s University of London, UK	2009	5	73	49	Saltar N and Seshasai SRK (5)
Rothwell, Peter M	University of Oxford, UK	2003	5	36	19	Belch JFF and Meade TW (3)
Seshasai, Sreenivasa RK	University of Cambridge, UK	2009	5	73	49	Ray KK and SN (5)
Simes, John	National Health and Medical Research Council Clinical Trials Centre, Australia	2002	5	53	36	Baigent C, Blackwell L, Collins R, Keech A and Peto R (3)
Smith, George Davey	University of Bristol, UK	1993	5	63	54	Ebrahim S (4)
Torgerson, David J	University of York, UK	1999	5	46	39	Adamson SJ and Bell Syer SEM (2)
Wetterslev, Jorn	Copenhagen University Hospital, Denmark	2007	5	28	15	Gluud C (3)
Wilt, Timothy J	Minneapolis VA Center for Chronic Disease Outcomes Research, USA	1998	5	40	32	MacDonald R (4)

^a^Joseph Lau was based at Tufts University Medical Center in Boston (USA) until 2012, and the institutional networks represent this fact.

**Figure 8:**
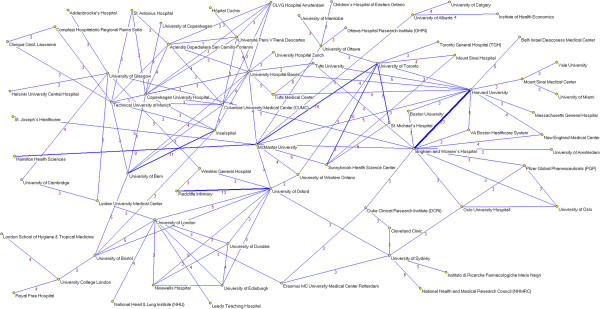
**Institutional networks.** Most productive cluster of institutions applying a threshold of three or more papers signed in co-authorship.

**Figure 9:**
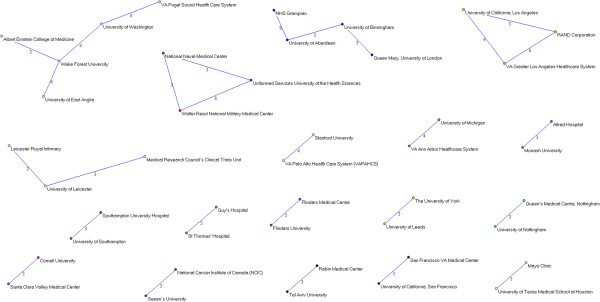
**Institutional networks.** Other relevant clusters of institutions applying a threshold of three or more papers signed in co-authorship.

Institutional productivity was headed by McMaster University (49 papers), University of Oxford (48 papers) and Harvard University (36 papers) (Table 3). Next came some of their affiliated hospitals or medical centers (Brigham and Women’s Hospital and the Radcliffe Infirmary, with 32 and 30, respectively). Applying a collaboration threshold of at least 3 papers signed with inter-institutional collaboration, we identified 19 clusters comprising a total of 120 institutions (Figures 8 and 9). Of these, the most important institutional cluster comprised 76 members.

**Table 3:** Ranking of most prolific institutions (≥ 10 papers) and their collaborative patterns

**Institution, country**	**Total papers, n**	**Signatures, n**	**Institutional collaborations (with different institutions), n**	**Institutional collaborations in same country (domestic institutions), n**	**Institutional collaborations from different countries, n**	**Main institutional collaborator (number of papers)**	**Different collaborative countries, n**
McMaster University, Canada	49	216	109	31	78	University of Toronto, Canada (10)	19
University of Oxford, UK	48	226	123	39	84	Radcliffe Infirmary, UK (13)	20
Harvard University, USA	36	210	104	47	57	Brigham and Women’s Hospital, USA (23)	16
Brigham and Women’s Hospital, USA	32	207	102	46	56	Harvard University, USA (23)	17
Radcliffe Infirmary, UK	30	96	46	14	32	University of Oxford, UK (13)	11
University of London, UK	29	207	125	34	91	University of Bristol, UK; University of Edinburgh, UK and University of Oxford, UK (5)	22
Copenhagen University Hospital, Denmark	28	169	93	8	85	Azienda Ospedaliera San Camillo-Forlanini, Italy; Université Paris V - René Descartes, France and University of Copenhagen, Denmark (4)	20
University of Toronto, Canada	27	123	55	23	32	McMaster University, Canada and Sunnybrook Health Science Center, Canada (4)	12
University of Sydney, Australia	23	120	70	14	56	National Health and Medical Research Council (NHMRC), Australia (6)	15
University of Birmingham, UK	22	80	51	26	25	Queen Mary, University of London, UK and University of Aberdeen, UK	(3) 11
University of Bristol, UK	22	141	96	33	63	University of London, UK (5)	21
University of Alberta, Canada	20	94	57	14	43	Institute of Health Economics, Canada and University of Calgary, Canada (4)	15
University College London, UK	19	123	88	33	55	London School of Hygiene & Tropical Medicine, UK and Royal Free Hospital, UK (4)	20
University Hospital Basel, Switzerland	18	185	113	9	104	Inselspital, Switzerland (5)	20
Mayo Clinic, USA	18	157	130	35	95	University of Texas Medical School at Houston, USA (3)	23
University of Glasgow, UK	17	124	85	32	53	University of Cambridge, UK (4)	14
Erasmus MC University Medical Center Rotterdam, the Netherlands	17	145	106	8	98	Duke Clinical Research Institute (DCRI), USA and University of Sydney, Australia (3)	23
Cleveland Clinic, UK	16	75	51	11	40	Duke Clinical Research Institute (DCRI), USA and University of Sydney, Australia (3)	14
The University of York, UK	16	64	44	26	18	University of Leeds, UK (3)	8
University of Bern, Switzerland	16	128	72	8	64	Inselspital, Switzerland (11)	16
Medical Research Council’s Clinical Trials Unit, UK	16	81	59	25	34	University of Leicester, UK (3)	14
Johns Hopkins University, USA	15	58	42	18	24	University Hospital Basel, Switzerland (2)	11
Instituto di Ricerche Farmacologiche Mario Negri, Italy	15	85	63	9	54	University of Sydney, Australia (3)	14
Hamilton Health Sciences, Canada	14	64	37	5	32	McMaster University, Canada (9)	11
University of Calgary, Canada	14	62	35	17	18	University of Alberta, Canada (4)	10
Tufts University, USA	14	79	45	14	31	Tufts Medical Center, USA (6)	13
University of California, San Francisco, USA	14	77	56	24	32	San Francisco VA Medical Center, USA (3)	16
University of Aberdeen, UK	13	73	50	25	25	NHS Grampian, UK (5)	11
Sunnybrook Health Science Center, Canada	13	83	45	17	28	University of Toronto, Canada (10)	9
University of Ottawa, Canada	13	70	40	16	24	Children’s Hospital of Eastern Ontario, Canada; McMaster University, Canada; Ottawa Hospital Research Institute (OHRI), Canada; University of Manitoba, Canada and University of Toronto, Canada (3)	13
Wake Forest University, USA	12	55	31	17	14	University of East Anglia, UK and University of Washington, USA (4)	7
Inselspital, Switzerland	12	119	67	8	59	University of Bern, Switzerland (11)	16
University of Washington, USA	12	44	25	12	13	VA Puget Sound Health Care System, USA and Wake Forest University, USA (4)	8
University of Edinburgh, UK	12	106	66	30	36	University of London, UK (5)	17
Technical University of Munich, Germany	11	110	63	3	60	Columbia University Medical Center (CUMC), USA (4)	17
Queen Mary, University of London, UK	11	46	31	8	23	University of Birmingham, UK (3)	9
Tufts Medical Center, USA	11	41	16	8	8	Tufts University, USA (6)	3
London School of Hygiene & Tropical Medicine, UK	11	106	86	15	71	University College London, UK (4)	25
University of Pittsburgh, USA	11	64	51	16	35	Erasmus MC University Medical Center Rotterdam, the Netherlands and Ohio State University, USA (2)	12

**Figure 10:**
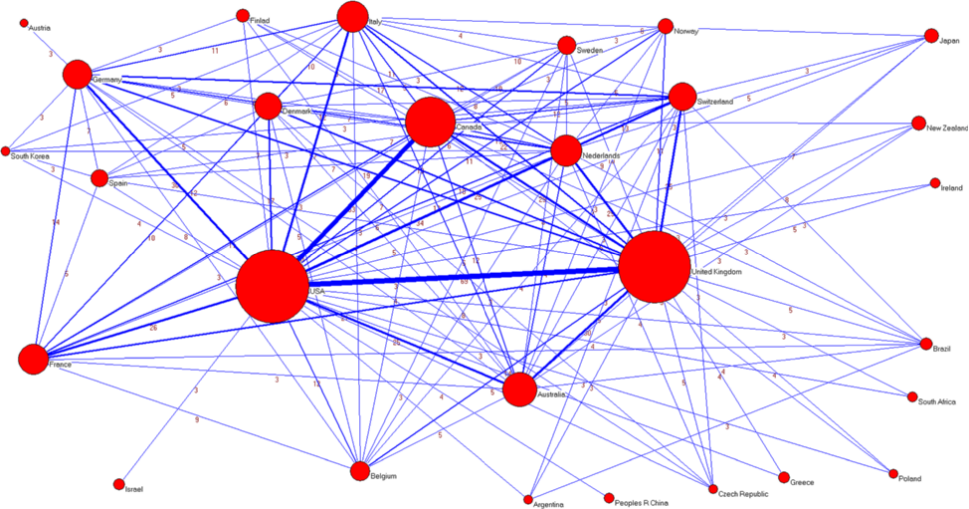
**Global collaborative network between countries.** Note: Node sizes are proportional to the number of papers and line thicknesses are proportional to the number of collaborations.

The productivity ranking for countries with respect to the number of papers (Table 4) was headed by the USA (310 papers), the UK (297 papers) and Canada (143 papers). After these countries came Australia (70 papers), and Italy and the Netherlands (57 papers each). The USA and the UK also headed the list of the number of different countries with which they had collaborated, as well as the total number of collaborations. Figure 10 shows a visual representation of the collaborative network between countries, in which we can see the relationships of some with respect to others and the position that each occupies in the network as a whole.

**Table 4:** Productivity and patterns of collaboration by countries

**Country**	**Total papers, n**	**Papers per million inhabitants, n**	**Collaborations, n**	**Distinct countries of collaborations, n**	**Main collaborator(s) (number of collaborations)**
USA	310	0.99	428	43	UK (63)
UK	297	4.73	380	44	USA (69)
Canada	143	4.15	200	29	USA (53)
Australia	70	3.14	165	30	UK (30)
Italy	57	0.94	205	29	USA (34)
The Netherlands	57	3.41	173	27	USA (27)
France	51	0.78	159	27	USA (26)
Germany	50	0.61	164	32	USA (30)
Switzerland	47	5.94	177	35	USA (25)
Denmark	41	7.36	124	28	UK (18)
Belgium	23	2.09	92	23	UK (14)
Sweden	21	2.22	69	14	USA (13)
Spain	19	0.41	76	28	USA (8)
Norway	14	2.83	56	20	UK and USA (11)
Japan	13	0.10	51	21	USA (9)
New Zealand	13	2.95	24	11	UK (8)
Finlad	11	2.04	43	14	Australia and USA (7)
Brazil	9	0.05	40	16	UK and USA (5)
Greece	8	0.71	12	5	UK and USA (4)
Israel	8	1.03	7	5	USA (3)
Ireland	6	1.31	24	14	UK (5)
China	6	0.00	10	7	USA (4)
South Africa	6	0.12	23	16	USA (4)
Argentina	5	0.12	24	13	Brazil, Switzerland, UK and USA (3)
Czech Republic	5	0.48	42	18	UK and USA (5)
Poland	5	0.13	23	13	UK (4)
South Korea	5	0.10	31	13	USA (4)
Austria	4	0.47	9	6	Germany (3)
Thailand	4	0.06	10	10	All countries (1)
Chile	3	0.17	10	8	Canada and USA (2)
India	3	0.00	2	2	Australia and UK (1)
Portugal	3	0.28	1	1	Canada (1)
Taiwan	3	0.13	3	3	Denmark, UK and USA (1)
Costa Rica	2	0.42	8	6	Canada and USA (2)
Hungary	2	0.20	16	14	UK and USA (2)
Pakistan	2	0.01	2	1	UK (2)
Russia	2	0.01	18	14	Belgium, Denmark, Germany, and UK (2)
Saudi Arabia	2	0.07	7	6	UK (2)
Serbia and Montenegro	2	0.28	4	2	Denmark and Italy (2)
Turkey	2	0.03	13	9	Australia, Canada, Switzerland and USA (2)
Colombia	1	0.02	6	6	All countries (1)
Cuba	1	0.09	5	5	All countries (1)
Gabon	1	0.65	10	10	All countries (1)
Ghana	1	0.04	10	10	All countries (1)
Latvia	1	0.49	12	12	All countries (1)
Mexico	1	0.01	1	1	USA (1)
Morocco	1	0.03	10	10	All countries (1)
Mozambique	1	0.04	10	10	All countries (1)
Republic of Malawi	1	0.07	1	1	UK (1)
Singapore	1	0.19	0	0	-
Tanzania	1	0.02	10	10	All countries (1)

Note: Country inhabitants (year 2011) were obtained from the World Bank.

The 75 most cited articles by number of citations are listed in the supplementary material (see Additional file 2: ‘List of most cited meta-analyses’). Heavily cited meta-analyses include randomized trials examining the health effects of pharmacological interventions in cardiology and oncology (for example, antithrombotic trials, anti-platelet trials, antihypertensive trials, lipid-lowering trials, chemotherapy for diverse cancers such as breast cancer, lung cancer, or head and neck cancers).

## Discussion

Generally, the visibility and recognition of scientific research activities benefits from increasing collaborative research [18]. Research collaboration plays an important role in science, policy and medicine [19,20]. Research collaboration in the ‘Big Science’ era involves addressing important and relevant research questions that require a complex construction of multi-disciplinary teams of scientists and researchers, large-scale scientific structures, budgets of an unprecedented scale, and widespread sharing of scientific knowledge and data. Thus, meta-analysis can be considered a good example of ‘Big Science’ in medicine and clinical epidemiology [21], as quantitative evidence synthesis is the application, in practice, of the principle that science is cumulative [22,23]. An obvious manifestation of this is the observed trend of 75 new randomized trials and 11 new systematic reviews being published daily, with a plateau in this growth not yet reached [24]. Therefore, promoting research collaboration in evidence synthesis is able to strengthen research activity, productivity and impact.

In general, we found a strong clustering of papers published in two British journals (*BMJ* and *The Lancet* accounted for 57% of meta-analyses), in contrast to other general medical journals (for example, the *NEJM* represented less than 2%). We hypothesized that these different findings between journals may potentially reflect an editorial policy and/or preference, with the *BMJ*, *The Lancet* and *JAMA* journals specifically being more interested in and/or promoting the publication of high-quality quantitative evidence synthesis.

Perhaps a relevant finding is that collaborative networks are expanding in multiple regions, revealing a discernable and well-established scientific community, with the most prolific authors and institutions having an important number of collaborations. As might be expected, the scientific community captured by the networks is centered on a nucleus of scientists and researchers from academia, medical centers and health research institutes from western high-income countries (North America, Western Europe and Australia/Oceania). Specifically, the most intense global collaborations took place between authors and institutions from the USA, the UK and Canada. However, although these three countries lead in the number of published high impact meta-analyses, the efforts during the period of study were global, with publications from authors and institutions in more than 50 different countries. Cultural links may have historically benefited some countries through alliances with nations and regions that speak the same language (as may be the case for the UK through alliances with Commonwealth countries that speak English) and have adopted similar scientific and research structures [25]. However, there is a clear over-representation of scientists based in western high-income countries, and the limited participation of low and middle income-based researchers could warrant further pragmatic action. Given that research resources and funding are often restricted, it is the responsibility of the scientific community to utilize the resources available most efficiently when exploring research priorities to afford the health needs of the population, stimulating north–south and west–east collaborations where possible. In fact, these results are consistent with those reported by Uthman *et al*. [26], who assessed the characteristics of the 100 most frequently cited meta-analysis related articles. Although the scope of our research is definitely different from that paper, those authors also showed that the USA, the UK and Canada have taken leadership in the production of citation papers, but no first author from low or middle income countries led one of the most cited papers.

The maps of scientific partnership show that authors who are ‘leaders’ and thus who may contribute collaboration, have more frequent and intense collaboration between other authors and institutions from different countries. The study also identifies highly cohesive cluster networks and provides considerable information on the structure that can be put to various purposes, such as funding agencies designing strategies for future scientific collaboration, agencies such as the World Health Organization promoting a global coordinated agenda for perceived high priority clinical topics, and sharing of reliable and innovative methodologies that can be linked to world-class educational and training opportunities.

There are several possible explanations for our findings. The use of modern communication and information technologies, especially the Internet, has diminished the role of geographical and territorial boundaries in the access and transmissibility of information [27]. This has enabled scientists, and particularly systematic reviewers, closer internalization of research and collaboration. Similarly, the creation of some international collaborations, including those conducting clinical trials, may have settled the groundwork for the subsequent realization of collaborative meta-analyses that may have a clear scientific and clinical impact. For example, according to SCI-E, the most cited meta-analysis article has received more than 2,500 citations; this was a paper by the Antithrombotic Trialists’ Collaboration [28] that contributed to determine the protective effects of anti-platelet therapy (such as low dose aspirin) for patients at high risk of occlusive vascular events. Analyses of the Oxford-based Early Breast Cancer Trialists Collaborative Group (EBCTCG) provided breakthrough examples of complete pictures of the evidence on the long-term effects of various therapies on early breast cancer [29-31].

Collaborative networks, an important form of social network analysis, have been intensively studied in many scientific disciplines, including biology, physics, medicine and economics [13,16,32-36]. To our knowledge, no study has previously described and characterized the global collaborative patterns and networks of published meta-analyses of randomized trials. Very few studies have reviewed evidence synthesis for decision-making using a research collaboration approach [35,36], and although not directly comparable with our analysis, there are aspects worthy of comment. A recent paper by Wagstaff and Culyer [35] examined four decades of health economics research. They compared authors, institutions, countries and journals in terms of the volume of publications in the US, the UK and Canada; Harvard University, the World Bank and the MIT emerged at the top on a variety of measures. Previously, Greenberg *et al*. [36] conducted a review of cost-effectiveness analyses of the English-language articles indexed in PubMed since 2006, and observed that the most prolific authors were affiliated with renowned US institutions in the USA (for example, Harvard University, Stanford University and Tufts University, and their affiliated hospitals).

There are several limitations to our study. First, although the scientific production analysed has been drawn from an exhaustive analysis of the literature, it is possible that the search missed some relevant articles. Furthermore, some reports were published in journals without being indexed as meta-analyses, making them difficult to identify. The analyses inevitably represent an initial investigation, and a more detailed exploration is also needed. In addition, we restricted our analysis to meta-analyses that considered randomized trials as the primary source of clinical evidence, and therefore there may be scientists and researchers (or institutions) who do not appear because their papers are not reflected in the collaborative networks (for example, genetic epidemiology). It would be interesting to explore whether the use of alternative sources (such as observational research, descriptive epidemiology, molecular genetics, non-clinical studies) resulted in similar results to those reported here. Our analysis was also limited in scope, focusing only on original research and reviews articles. Undoubtedly, there are other important reports (for example, methodological [37-39] and conceptual papers [6,40,41]) that also merit consideration. Second, we excluded the Cochrane Library*,* specifically, the Cochrane Database of Systematic Reviews, a major source of systematic reviews. However, to date its impact factor is smaller than that of any of the included journals. Given the dynamic nature of the field, other opportunities for further research include examining the evolution of the identified networks over time (for example, by means of longitudinal analysis) also considering papers published in multi-disciplinary journals or those included in journals belonging to other categories.

Additionally, there were no further inquiries or attempts to verify the quality of reporting of the meta-analyses in our sample. Previous research [42] has addressed this issue, pointing out that some of the meta-analyses published in leading medical journals have important methodological limitations. Third, as in many bibliometric analyses, the importance of normalizing the names of scientists, researchers and their institutions is fundamental to avoiding potential errors caused in recognizing variations in the name of a single author. Nevertheless, we conducted a careful manual validation of the bibliographic references to avoid these potential errors. In the case of authors, the criterion followed with two or more variants of a name or surname was to check the coincidence of the different variants with the workplace. As discussed elsewhere [34], this procedure does not assure complete certainty. It does not take into account possible changes in the author’s workplace, nor does it avoid the problem where the same bibliographic name refers to the scientific production of two or more authors, although the fact that a single field and a short chronologic period were being analyzed helped to minimize this kind of error. For institutional names, the main problem is that the same name frequently applies to two or more institutions, something that is common for authors who work in institutes or hospitals connected to universities. In such cases, we opted to assign as many names to the macroinstitutions as could be identified. Although this resulted in the problem of multiplying the number of institutions in the recount, it was necessary in order to avoid losing information concerning the macroinstitutions occurring in second place or later in the list of names. The same criterion of multiplying the names was used in the case of the institutes and other research organizations, sometimes administratively dependent on one macroinstitution, the result being that a ‘fictitious’ inter-institutional collaboration may have been obtained.

## Conclusions

Our study identified the most significant collaborative networks of authors, institutions and countries publishing meta-analyses of randomized trials in high impact medical journals. This information may be used to strengthen scientific capacity for global collaboration and help to build a cooperative scientific agenda for future research of excellence in the field of clinical evidence synthesis in a manner similar to how some international clinical trial collaborations have developed. We hope that this analysis will be useful as policy makers, researchers and institutions look to the future.

## Competing interests

The authors declare that they have no competing interest.

## Authors’ contributions

FC-L conceived the original idea. AA-A, RA-B and FC-L designed the study. AA-A and FC-L collected the data and performed the statistical analysis. FC-L and AA-A take responsibility for the integrity of the data and the accuracy of the data analysis. DM, BH and FC-L interpreted the data. All authors wrote and/or critically revised the manuscript for important intellectual content. All authors approved the final version of the manuscript. FC-L is the guarantor.

## Author’s information

RA-B and DM are joint senior authors.

## Pre-publication history

The pre-publication history for this paper can be accessed here:

http://www.biomedcentral.com/1741-7015/12/15/prepub

## Supplementary Material

Additional file 1 Definitions of collaborative measurements. Click here for file

Additional file 2 List of most cited meta-analyses. Click here for file
