# Supplementary material for: Global collaborative networks on meta-analyses of randomized trials published in high impact factor medical journals: a social network analysis
Source: BMC Med. 2014 Jan 29;12:15. doi: 10.1186/1741-7015-12-15 (PMC3913337; doi:10.1186/1741-7015-12-15)
Supplement: Additional file 1 — Definitions of collaborative measurements. [file 1741-7015-12-15-S1.doc]

**Additional file 1: Definitions of the measurements of collaboration.**

| **Measure** | **Definition** |
| --- | --- |
| *Number of papers* | Number of published articles included in the survey sample |
| *Number of signatures* | Total number of authors included in all the papers of each author |
| *Number of collaborations* | Number of different authors included in all the papers of each author |
| *Index of signatures per paper (collaboration index)* | Mean number of signatures per paper |
| *Index of authors per paper* | Mean number of authors per paper considering only the different authors |
