# Supplementary material for: Global collaborative networks on meta-analyses of randomized trials published in high impact factor medical journals: a social network analysis
Source: BMC Med. 2014 Jan 29;12:15. doi: 10.1186/1741-7015-12-15 (PMC3913337; doi:10.1186/1741-7015-12-15)
Supplement: Additional file 2 — List of most cited meta-analyses. [file 1741-7015-12-15-S2.doc]

**Additional file 2: List of most cited meta-analyses.**

| **Reference (main authors and title)** | **Journal** | **Year** | **Total citations** | **Citations/ Year since publication** |
| --- | --- | --- | --- | --- |
| **Antithrombotic Trialists' Collaboration. Collaborative meta-analysis of randomised trials of antiplatelet therapy for prevention of death, myocardial infarction, and stroke in high risk patients.** | **BMJ** | **2002** | **2686** | **269** |
| **Early Breast Cancer Trialists' Collaborative Group (EBCTCG). Effects of chemotherapy and hormonal therapy for early breast cancer on recurrence and 15-year survival: an overview of the randomised trials.** | **Lancet** | **2005** | **2417** | **345** |
| **Antiplatelet Trialists' Collaboration. Collaborative overview of randomised trials of antiplatelet therapy-I: Prevention of death, myocardial infarction, and stroke by prolonged antiplatelet therapy in various categories of patients.** | **BMJ** | **1994** | **2331** | **130** |
| **Non-small Cell Lung Cancer Collaborative Group. Chemotherapy in non-small cell lung cancer: a meta-analysis using updated data on individual patients from 52 randomised clinical trials.** | **BMJ** | **1995** | **2099** | **123** |
| **Baigent C, Keech A, Kearney PM, et al. (Cholesterol Treatment Trialists' Collaborators). Efficacy and safety of cholesterol-lowering treatment: prospective meta-analysis of data from 90,056 participants in 14 randomised trials of statins.** | **Lancet** | **2008** | **2092** | **523** |
| **Nissen SE, Wolski K. Effect of rosiglitazone on the risk of myocardial infarction and death from cardiovascular causes.** | **New Engl J Med** | **2007** | **1532** | **306** |
| **[No authors listed]. Risk factors for stroke and efficacy of antithrombotic therapy in atrial fibrillation. Analysis of pooled data from five randomized controlled trials.** | **Arch Intern Med** | **1994** | **1352** | **75** |
| **Early Breast Cancer Trialists' Collaborative Group. Polychemotherapy for early breast cancer: an overview of the randomised trials.** | **Lancet** | **1998** | **1318** | **94** |
| **Moher D, Pham B, Jones A, et al. Does quality of reports of randomised trials affect estimates of intervention efficacy reported in meta-analyses?** | **Lancet** | **1998** | **1285** | **92** |
| **Pignon JP, Bourhis J, Domenge C, Designé L. Chemotherapy added to locoregional treatment for head and neck squamous-cell carcinoma: three meta-analyses of updated individual data. MACH-NC Collaborative Group. Meta-Analysis of Chemotherapy on Head and Neck Cancer.** | **Lancet** | **2000** | **1176** | **98** |
| **Garg R, Yusuf S. Overview of randomized trials of angiotensin-converting enzyme inhibitors on mortality and morbidity in patients with heart failure. Collaborative Group on ACE Inhibitor Trials.** | **JAMA** | **1995** | **1032** | **61** |
| **Turnbull F; Blood Pressure Lowering Treatment Trialists' Collaboration. Effects of different blood-pressure-lowering regimens on major cardiovascular events: results of prospectively-designed overviews of randomised trials** | **Lancet** | **2003** | **1004** | **112** |
| **Hacke W, Donnan G, Fieschi C, et al. Association of outcome with early stroke treatment: pooled analysis of ATLANTIS, ECASS, and NINDS rt-PA stroke trials** | **Lancet** | **2004** | **983** | **123** |
| **Mukherjee D, Nissen SE, Topol EJ. Risk of cardiovascular events associated with selective COX-2 inhibitors.** | **JAMA** | **2001** | **967** | **88** |
| **Bongartz T, Sutton AJ, Sweeting MJ, Buchan I, Matteson EL, Montori V. Anti-TNF antibody therapy in rheumatoid arthritis and the risk of serious infections and malignancies: systematic review and meta-analysis of rare harmful effects in randomized controlled trials.** | **JAMA** | **2006** | **859** | **143** |
| **Antman EM, Lau J, Kupelnick B, Mosteller F, Chalmers TC. A comparison of results of meta-analyses of randomized control trials and recommendations of clinical experts. Treatments for myocardial infarction.** | **JAMA** | **1992** | **786** | **39** |
| **Miller ER 3rd, Pastor-Barriuso R, Dalal D, Riemersma RA, Appel LJ, Guallar E. Meta-analysis: high-dosage vitamin E supplementation may increase all-cause mortality.** | **Ann Intern Med** | **2005** | **776** | **111** |
| **Law MR, Wald NJ, Thompson SG. By how much and how quickly does reduction in serum cholesterol concentration lower risk of ischaemic heart disease?** | **BMJ** | **1994** | **772** | **43** |
| **Wong DK, Cheung AM, O'Rourke K, Naylor CD, Detsky AS, Heathcote J. Effect of alpha-interferon treatment in patients with hepatitis B e antigen-positive chronic hepatitis B. A meta-analysis.** | **Ann Intern Med** | **1993** | **755** | **40** |
| **Stone GW, Moses JW, Ellis SG, et al. Safety and efficacy of sirolimus- and paclitaxel-eluting coronary stents.** | **New Engl J Med** | **2007** | **740** | **148** |
| **Hart RG, Benavente O, McBride R, Pearce LA. Antithrombotic therapy to prevent stroke in patients with atrial fibrillation: a meta-analysis.** | **Ann Intern Med** | **1999** | **725** | **56** |
| **Neal B, MacMahon S, Chapman N; Blood Pressure Lowering Treatment Trialists' Collaboration. Effects of ACE inhibitors, calcium antagonists, and other blood-pressure-lowering drugs: results of prospectively designed overviews of randomised trials.** | **Lancet** | **2000** | **716** | **60** |
| **Homocysteine Lowering Trialists' Collaboration. Lowering blood homocysteine with folic acid based supplements: meta-analysis of randomised trials.** | **BMJ** | **1998** | **701** | **50** |
| **LaRosa JC, He J, Vupputuri S. Effect of statins on risk of coronary disease: a meta-analysis of randomized controlled trials.** | **JAMA** | **1999** | **688** | **53** |
| **Linde K, Ramirez G, Mulrow CD, Pauls A, Weidenhammer W, Melchart D. St John's wort for depression--an overview and meta-analysis of randomised clinical trials.** | **BMJ** | **1996** | **664** | **42** |
| **LeLorier J, Grégoire G, Benhaddad A, Lapierre J, Derderian F. Discrepancies between meta-analyses and subsequent large randomized, controlled trials.** | **New Engl J Med** | **1997** | **657** | **44** |
| **Bjelakovic G, Nikolova D, Gluud LL, Simonetti RG, Gluud C. Mortality in randomized trials of antioxidant supplements for primary and secondary prevention: systematic review and meta-analysis** | **JAMA** | **2008** | **641** | **160** |
| **Lau J, Antman EM, Jimenez-Silva J, Kupelnick B, Mosteller F, Chalmers TC. Cumulative meta-analysis of therapeutic trials for myocardial infarction.** | **New Engl J Med** | **1992** | **635** | **32** |
| **Flather MD, Yusuf S, Køber L, et al. Long-term ACE-inhibitor therapy in patients with heart failure or left-ventricular dysfunction: a systematic overview of data from individual patients. ACE-Inhibitor Myocardial Infarction Collaborative Group.** | **Lancet** | **2000** | **621** | **52** |
| **Weaver WD, Simes RJ, Betriu A, et al. Comparison of primary coronary angioplasty and intravenous thrombolytic therapy for acute myocardial infarction: a quantitative review.** | **JAMA** | **1997** | **620** | **41** |
| **Mauri L, Hsieh WH, Massaro JM, Ho KK, D'Agostino R, Cutlip DE. Stent thrombosis in randomized clinical trials of drug-eluting stents.** | **New Engl J Med** | **2007** | **615** | **123** |
| **Early Breast Cancer Trialists' Collaborative Group. Effects of radiotherapy and surgery in early breast cancer. An overview of the randomized trials.** | **New Engl J Med** | **1995** | **592** | **35** |
| **Province MA, Hadley EC, Hornbrook MC, et al. The effects of exercise on falls in elderly patients. A preplanned meta-analysis of the FICSIT Trials. Frailty and Injuries: Cooperative Studies of Intervention Techniques.** | **JAMA** | **1995** | **575** | **34** |
| **Wald NJ, Law MR. A strategy to reduce cardiovascular disease by more than 80%.** | **BMJ** | **2003** | **574** | **64** |
| **Law MR, Wald NJ, Rudnicka AR. Quantifying effect of statins on low density lipoprotein cholesterol, ischaemic heart disease, and stroke:systematic review and meta-analysis.** | **BMJ** | **2003** | **562** | **62** |
| **Stewart LA. Chemotherapy in adult high-grade glioma: a systematic review and meta-analysis of individual patient data from12 randomised trials.** | **Lancet** | **2002** | **549** | **55** |
| **Bischoff-Ferrari HA, Dawson-Hughes B, Willett WC, et al. Effect of Vitamin D on falls: a meta-analysis.** | **JAMA** | **2004** | **540** | **68** |
| **Rothwell PM, Eliasziw M, Gutnikov SA, et al. Analysis of pooled data from the randomised controlled trials of endarterectomy for symptomatic carotid stenosis.** | **Lancet** | **2003** | **539** | **60** |
| **Freemantle N, Cleland J, Young P, Mason J, Harrison J. beta Blockade after myocardial infarction: systematic review and meta regression analysis.** | **BMJ** | **1999** | **539** | **41** |
| **Bischoff-Ferrari HA, Willett WC, Wong JB, Giovannucci E, Dietrich T, Dawson-Hughes B. Fracture prevention with vitamin D supplementation: a meta-analysis of randomized controlled trials.** | **JAMA** | **2005** | **535** | **76** |
| **Kasiske BL, Kalil RS, Ma JZ, Liao M, Keane WF. Effect of antihypertensive therapy on the kidney in patients with diabetes: a meta-regression analysis.** | **Ann Inter Med** | **1993** | **535** | **28** |
| **Cochrane Injuries Group Albumin Reviewers. Human albumin administration in critically ill patients: systematic review of randomised controlled trials.** | **BMJ** | **1998** | **534** | **38** |
| **Psaty BM, Smith NL, Siscovick DS, et al. Health outcomes associated with antihypertensive therapies used as first-line agents. A systematic review and meta-analysis.** | **JAMA** | **1997** | **533** | **36** |
| **Aupérin A, Arriagada R, Pignon JP, et al. Prophylactic cranial irradiation for patients with small-cell lung cancer in complete remission. Prophylactic Cranial Irradiation Overview Collaborative Group.** | **New Engl J Med** | **1999** | **524** | **40** |
| **Maggard MA, Shugarman LR, Suttorp M, et al. Meta-analysis: surgical treatment of obesity.** | **Ann Intern Med** | **2005** | **517** | **74** |
| **Kearney PM, Baigent C, Godwin J, Halls H, Emberson JR, Patrono C. Do selective cyclo-oxygenase-2 inhibitors and traditional non-steroidal anti-inflammatory drugs increase the risk of atherothrombosis? Meta-analysis of randomised trials.** | **BMJ** | **2006** | **512** | **85** |
| **Rothwell PM, Eliasziw M, Gutnikov SA, Warlow CP, Barnett HJ; Carotid Endarterectomy Trialists Collaboration. Endarterectomy for symptomatic carotid stenosis in relation to clinical subgroups and timing of surgery.** | **Lancet** | **2004** | **511** | **64** |
| **Humphrey LL, Helfand M, Chan BK, Woolf SH. Breast cancer screening: a summary of the evidence for the U.S. Preventive Services Task Force.** | **Ann Intern Med** | **2002** | **499** | **50** |
| **Jafar TH, Schmid CH, Landa M, et al. Angiotensin-converting enzyme inhibitors and progression of nondiabetic renal disease. A meta-analysis of patient-level data.** | **Ann Intern Med** | **2001** | **498** | **45** |
| **Stettler C, Wandel S, Allemann S, et al. Outcomes associated with drug-eluting and bare-metal stents: a collaborative network meta-analysis.** | **Lancet** | **2007** | **491** | **98** |
| **Boersma E, Harrington RA, Moliterno DJ, et al. Platelet glycoprotein IIb/IIIa inhibitors in acute coronary syndromes: a meta-analysis of all major randomised clinical trials.** | **Lancet** | **2002** | **490** | **49** |
| **Kastrati A, Mehilli J, Pache J, et al. Analysis of 14 trials comparing sirolimus-eluting stents with bare-metal stents.** | **New Engl J Med** | **2007** | **481** | **96** |
| **Stroke Unit Trialists' Collaboration. Collaborative systematic review of the randomised trials of organised inpatient (stroke unit) care after stroke.** | **BMJ** | **1997** | **476** | **32** |
| **Shang A, Huwiler-Müntener K, Nartey L, et al. Are the clinical effects of homoeopathy placebo effects? Comparative study of placebo-controlled trials of homoeopathy and allopathy.** | **Lancet** | **2005** | **475** | **68** |
| **Staessen JA, Wang JG, Thijs L. Cardiovascular protection and blood pressure reduction: a meta-analysis.** | **Lancet** | **2001** | **472** | **43** |
| **Autier P, Gandini S. Vitamin D supplementation and total mortality: a meta-analysis of randomized controlled trials.** | **Arch Intern Med** | **2007** | **458** | **92** |
| **Langman MJ, Jensen DM, Watson DJ, et al. Adverse upper gastrointestinal effects of rofecoxib compared with NSAIDs.** | **JAMA** | **1999** | **455** | **35** |
| **Antithrombotic Trialists' (ATT) Collaboration, Baigent C, Blackwell L, et al. Aspirin in the primary and secondary prevention of vascular disease: collaborative meta-analysis of individual participant data from randomised trials.** | **Lancet** | **2009** | **448** | **149** |
| **Walsh BT, Seidman SN, Sysko R, Gould M. Placebo response in studies of major depression: variable, substantial, and growing.** | **JAMA** | **2002** | **448** | **45** |
| **Schneider LS, Dagerman KS, Insel P. Risk of death with atypical antipsychotic drug treatment for dementia: meta-analysis of randomized placebo-controlled trials.** | **JAMA** | **2005** | **441** | **63** |
| **Pearson DC, May GR, Fick GH, Sutherland LR. Azathioprine and 6-mercaptopurine in Crohn disease. A meta-analysis.** | **Ann Intern Med** | **1995** | **441** | **26** |
| **Ferrari MD, Roon KI, Lipton RB, Goadsby PJ. Oral triptans (serotonin 5-HT(1B/1D) agonists) in acute migraine treatment: a meta-analysis of 53 trials.** | **Lancet** | **2001** | **437** | **40** |
| **Whittington CJ, Kendall T, Fonagy P, Cottrell D, Cotgrove A, Boddington E. Selective serotonin reuptake inhibitors in childhood depression: systematic review of published versus unpublished data.** | **Lancet** | **2004** | **436** | **55** |
| **Mermel LA. Prevention of intravascular catheter-related infections.** | **Ann Intern Med** | **2000** | **436** | **36** |
| **Colorectal Cancer Collaborative Group. Adjuvant radiotherapy for rectal cancer: a systematic overview of 8,507 patients from 22 randomised trials.** | **Lancet** | **2001** | **435** | **40** |
| **Lincoff AM, Wolski K, Nicholls SJ, Nissen SE. Pioglitazone and risk of cardiovascular events in patients with type 2 diabetes mellitus: a meta-analysis of randomized trials.** | **JAMA** | **2007** | **427** | **85** |
| **Fiore MC, Smith SS, Jorenby DE, Baker TB. The effectiveness of the nicotine patch for smoking cessation. A meta-analysis.** | **JAMA** | **1994** | **427** | **24** |
| **Vivekananthan DP, Penn MS, Sapp SK, Hsu A, Topol EJ. Use of antioxidant vitamins for the prevention of cardiovascular disease: meta-analysis of randomised trials.** | **Lancet** | **2003** | **426** | **47** |
| **Schmieder RE, Martus P, Klingbeil A. Reversal of left ventricular hypertrophy in essential hypertension. A meta-analysis of randomized double-blind studies.** | **JAMA** | **1996** | **422** | **26** |
| **Law MR, Wald NJ, Morris JK, Jordan RE. Value of low dose combination treatment with blood pressure lowering drugs: analysis of 354 randomised trials.** | **BMJ** | **2003** | **416** | **46** |
| **Green JA, Kirwan JM, Tierney JF, et al. Survival and recurrence after concomitant chemotherapy and radiotherapy for cancer of the uterine cervix: a systematic review and meta-analysis.** | **Lancet** | **2001** | **412** | **37** |
| **Lindholm LH, Carlberg B, Samuelsson O. Should beta blockers remain first choice in the treatment of primary hypertension? A meta-analysis.** | **Lancet** | **2005** | **411** | **59** |
| **Bradley DJ, Bradley EA, Baughman KL, et al. Cardiac resynchronization and death from progressive heart failure: a meta-analysis of randomized controlled trials.** | **JAMA** | **2003** | **406** | **45** |
| **Jüni P, Nartey L, Reichenbach S, Sterchi R, Dieppe PA, Egger M. Risk of cardiovascular events and rofecoxib: cumulative meta-analysis.** | **The Lancet** | **2004** | **404** | **51** |
| **Turpie AG, Bauer KA, Eriksson BI, Lassen MR. Fondaparinux vs enoxaparin for the prevention of venous thromboembolism in major orthopedic surgery: a meta-analysis of 4 randomized double-blind studies.** | **Arch Intern Med** | **2002** | **403** | **40** |
